# Plasma levels of CD36 and glutathione as biomarkers for ruptured intracranial aneurysm

**DOI:** 10.1515/biol-2022-0757

**Published:** 2023-12-31

**Authors:** Hanbin Wang, Luxuan Wang, Yunmei Liu, Weidong Men, Wanjiao Hao, Chuan Fang, Chunhui Li, Lijian Zhang

**Affiliations:** Department of Neurosurgery, Affiliated Hospital of Hebei University, Hebei University, Baoding, 071000, Hebei Province, China; Department of Neurological Function Examination, Affiliated Hospital of Hebei University, Hebei University, Baoding, 071000, Hebei Province, China; Department of Reproductive Medicine, Affiliated Hospital of Hebei University, Hebei University, Baoding, 071000, Hebei Province, China; Postdoctoral Research Station of Neurosurgery, Affiliated Hospital of Hebei University, Hebei University, Baoding, 071000, Hebei Province, China; Key Laboratory of Precise Diagnosis and Treatment of Glioma in Hebei Province, Affiliated Hospital of Hebei University, Hebei University, Baoding, 071000, Hebei Province, China

**Keywords:** intracranial aneurysm, inflammation, oxidative stress, CD36, glutathione, biomarker

## Abstract

Evidence has proved that intracranial aneurysm (IA) formation and rupture might be closely related to inflammatory response and oxidative stress. Our objective was to evaluate the potential of CD36 and glutathione (GSH) as biomarkers for IA. In this study, the enzyme-linked immunosorbent assay was used to measure the plasma levels of CD36 and GSH in 30 IA patients and 30 healthy controls. Then, correlation analysis, receiver operating characteristic (ROC) curve, and logistic regression analysis were performed. The results showed that the plasma level of CD36 in IA patients was significantly higher than that in the control group (*P* < 0.0001), and plasma GSH was significantly lower compared with that in the control group (*P* < 0.0001). ROC analysis showed that CD36 and GSH had high sensitivity (90.0 and 96.6%) and specificity (96.6 and 86.6%) for IA diagnosis. The combined sensitivity and specificity achieved were 100 and 100%, respectively. The plasma levels of CD36 and GSH did not show a significant correlation with age, the Glasgow Coma Scale, Hunter–Hess score, aneurysm size, aneurysm height, aneurysm neck, and aspect ratio. The AUC of the logistic regression model based on CD36 and GSH was 0.505. Our results suggested that the combination of plasma CD36 and GSH could serve as potential biomarkers for IA rupture.

## 1 Introduction

An intracranial aneurysm (IA) is an abnormal bulge of blood vessels caused by damage to the intracranial artery wall [1]. IA has an incidence of 3% in the general population. With the advances in medical imaging, an increasing number of patients are diagnosed with IA [2]. Patients with IA are usually accompanied by neurological dysfunction, and the mortality rate resulting from ruptured IA is as high as 50% [3,4]. Until now, the pathological mechanisms of IA formation and rupture remain unclear, which deserves deeper exploration for biomarker identification and therapeutic target discovery [5].

Previous studies have demonstrated that the formation and rupture of IA were closely related to several factors, including hemodynamics, inflammation, oxidative stress, endothelial dysfunction, and vascular remodeling [6,7]. Among them, inflammatory response and oxidative stress might be crucial pathophysiological processes for its occurrence, development, and rupture [8]. In order to identify new therapeutic targets and determine their prognostic roles to assist IA management, we selected a panel of inflammatory and oxidative stress-related markers, involved in vascular dysfunction. In this study, we focused on CD36 and glutathione (GSH), which are reported to participate in vascular remodeling under different pathological conditions of cardiovascular disease [9,10]. As a membrane glycoprotein, CD36 has been evidenced to play an essential role in physiological processes and cellular events such as inflammation, lipid metabolism, endothelial dysfunction, smooth muscle cell dysfunction, and foam cell formation [11]. GSH, a non-protein thiol, is involved in various redox reactions in the body and plays a vital role in protecting cells from oxidative damage and maintaining redox homeostasis [12,13].

Previous studies have documented that CD36 and GSH play crucial roles in processes such as inflammation and oxidative stress responses of arterial walls in cardiovascular diseases such as atherosclerosis [14,15]. CD36 expressed by macrophages promotes the inflammatory response and the formation of foam cells leading to the development of atherosclerosis [16]. The activation of inflammatory response and the upregulated expression of CD36 could also increase the production of reactive oxygen species (ROS), which would deplete GSH and trigger oxidative stress. Decreased GSH levels could further increase CD36 expression [17]. Recent studies have shown that IA is also a macrophage-mediated chronic inflammatory disease and that it may have atherosclerotic wall properties [18,19]. Here, we proposed that CD36 and GSH might play significant roles in IA rupture. To the best of our knowledge, however, there are no studies that have evaluated their expression profile in IA.

In this study, we measured the expression levels of plasma CD36 and GSH in IA patients and healthy controls (HCs) by using enzyme-linked immunosorbent assay (ELISA). We further investigated the correlation between clinical parameters of IA patients as well as CD36 and GSH expression levels. Further, we evaluated their potential utility as biomarkers for IA rupture.

## 2 Materials and methods

### 2.1 Study population

In this study, we recruited 30 IA patients from the Department of Neurosurgery, Affiliated Hospital of Hebei University. Inclusion criteria for the IA group were as follows: at least one ruptured IA found by digital subtraction angiography (DSA) or cerebral CT angiography (CTA) in patients with subarachnoid hemorrhage, no relevant symptoms, received non-operative and conservative observation treatment, and signing the informed consent. The exclusion criteria were as follows: the presence of intracranial tumors in patients with other cerebrovascular diseases (such as intracranial arteriovenous malformations, arteriovenous fistula, and Moyamoya syndrome/disease)and patients with myocardial infarction, pulmonary infarction, diabetes, metabolic syndrome, hepatitis, kidney diseases or progressive muscular atrophy, malignant tumor, leukemia, hemolytic anemia, etc. In parallel, another 30 age- and gender-matched healthy volunteers with no history of IA were included. Healthy volunteers underwent CTA before joining the control group, which recruited healthy volunteers with negative examination results. All protocols in this study were approved by the Institutional Review Board for human studies at the Affiliated Hospital of Hebei University, Hebei, China (HDFYLL-KY-2022-014). All participants signed informed consent of this study.


**Informed consent:** Informed consent has been obtained from all individuals included in this study.
**Ethical approval:** The research related to human use has complied with all the relevant national regulations, and institutional policies and in accordance with the tenets of the Helsinki Declaration, and has been approved by the Affiliated Hospital of Hebei University, Hebei, China (HDFYLL-KY-2022-014).

### 2.2 Standardized clinical assessment

**Figure 1:**
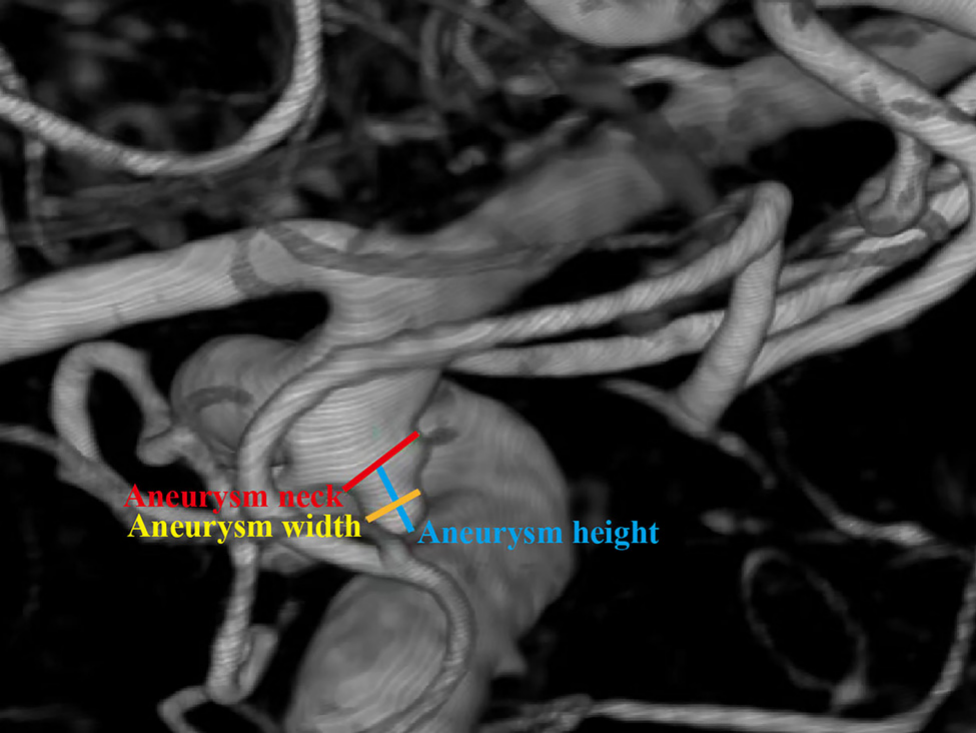
Definition of aneurysm parameters with representative image of DSA.

Standard clinical scores were employed in this study: neurological status was assessed using the Glasgow Coma Scale (GCS) and the Hunt–Hess scale. The lower the GCS score and the higher the Hunt–Hess scale grading, the worse the patient’s state of consciousness. Meanwhile, the demographics of all participants were collected. Aneurysm parameters include aneurysm location, aneurysm size, aneurysm height, aneurysm width, and aspect ratio (aneurysm height/aneurysm width) [20]. Aneurysm parameters were determined by analyzing the original and three-dimensional reconstruction images of the imaging examinations of CTA or DSA (Figure 1). The aneurysm height was measured as the maximum perpendicular distance between the dome and the neck plane [21]. The aneurysm width was measured as the maximal horizontal length of the aneurysm [22]. The aneurysm parameters were collected by two experienced neurosurgeons. All differences were resolved by consensus.

### 2.3 Blood sample collection

Blood samples were collected from patients within 12 h of admission. About 10 mL of venous blood from patients and healthy volunteers were collected, placed in EDTA tubes, stored at 4℃, and processed within 4 h. The venous blood was put into a centrifuge and centrifuged at 1,600*g* for 10 min at 4℃. The plasma obtained after centrifugation of the venous blood was then stored in a −80℃ freezer until further analysis [23].

### 2.4 Measurement of CD36 and GSH

The GSH and CD36 expression levels in the plasma of IA and HC were measured using ELISA as described previously [24,25]. We measured the concentration of sCD36 in plasma using ELISA kits (Nordic BioSite, Täby, Sweden). In the measurement of CD36, a pool of EDTA plasma was aliquoted, analyzed at seven different dilutions, and used as a standard concentration curve. Two dilutions of another EDTA pool served as internal controls, each of which was reproduced four times on each ELISA plate. According to the manufacturer’s instructions for the ELISA kit (A006-2-1, Nanjing Jian Cheng Bioengineering Institute, Nanjing, China), GSH levels were measured. Standards and samples were added to the wells precoated with GSH antigen. After incubation, the biotin-labeled anti-GSH antibody was added. The colorimetric reaction was initiated by the addition of yellow *para*-nitrophenylphosphatase (*p*NPP; Sigma) to each well, and absorbance values at 405 and 603 nm were measured on an ELISA microplate reader (Bio-Tek, Winooski, VT, USA).

### 2.5 Construction of the logistic regression model

Logistic regression is a classifier that uses a set of weighted measurements to predict the class (e.g., healthy, diseased) to which a sample belongs based on the probability. Moreover, the logistic regression is used to fit models for the probability of disease, given the marker values [26]. To investigate the reliability of GSH and CD36 as biomarkers for IA, the logistic regression models were constructed by using the glm function in R language. Logistic analysis can be used to predict the probability of an event. A model for predicting the occurrence of IA was established by logistic analysis combining clinical characteristics, aneurysm parameters, CD36, and GSH. In this logistic regression model, the expression values of GSH and CD36 were considered as predictive variables, and the clinical characteristics and aneurysm parameters, were considered as a binary responsive variable. A receiver operating characteristic (ROC) curve analysis was performed to evaluate the performance of the model.

### 2.6 Statistical analysis

All data were statistically analyzed and graphically displayed using GraphPad Prism 7.0 (GraphPad Software Inc., San Diego, CA, USA) and SPSS v.21.0 statistical packages (IBM, Armonk, NY, USA). The normal distribution of all data in this study was evaluated using the Shapiro–Wilk method (Table S1). Independent sample *t-*tests and Chi-square tests were used to compare the difference between baselines and stimuli tests. The values of CD36 and GSH expression levels did not conform to the normal distribution. Thus, the Mann–Whitney *U* test was used to analyze the difference in the CD36 and GSH expression between IA and control groups. Spearman correlation was applied to evaluate the relationship between the concentrations of GSH and CD36 expression, clinical data, and aneurysm parameters. The area under the ROC curve was employed to evaluate the potential diagnostic capability. The investigator who performed the statistical analysis was blind to the experimental groups. We repeated the analysis of the sample data. All values are expressed as mean ± SD. A *P*-value <0.05 was considered statistically significant.

## 3 Results

### 3.1 Demographic characteristics of the participants

There were 22 females and 8 males enrolled in the IA group, and the mean age of the IA patients was 56.2 ± 10.1 years (range 36–75). There were 20 females and 10 males in the HC group, and the mean age of the HC volunteers was 52.7 ± 11.6 years (range: 32–72). There was no age- and gender-related difference in the CD36 and GSH expression. The clinical data and aneurysm parameters of IA patients are presented in Table 1.

**Table 1:** Clinical data and aneurysm parameters of the study population

	HCs (*n* = 30)	IA patients (*n* = 30)
Gender (female/male)	20/10	22/8
Age, years	52.7 ± 11.6	56.2 ± 10.1
Smoking	**7**	6
Alcohol	**8**	**6**
Hypertension	**15**	19
**GCS**		
Mild (13–15 points)	—	22
Moderate (9–12 points)	—	8
Severe (3–8 points)	—	0
**Hunt**–**Hess grade**		
Ⅰ	—	4
Ⅱ	—	23
Ⅲ–Ⅴ	—	3
**Size of the aneurysm**		
<5 mm	—	15
5–10 mm	—	12
>10 mm	—	3
**Location of the aneurysm**		
Middle cerebral artery	—	10
Internal carotid artery	—	10
Anterior cerebral artery	—	2
Posterior cerebral artery	—	1
Anterior communicating artery	—	5
Posterior communicating artery	—	1
Basilar artery	—	1
Height of the aneurysm (mm)	—	5.1 ± 2.5 (range, 1.9–12.4)
Aneurysm neck (mm)	—	3.1 ± 1.5 (range, 1.2–7.3)

Bold value represents there was no difference in smoking between the healthy and IA groups (*P* = 0.754); alcohol between the healthy and IA groups (*P* = 0.542); hypertension between the healthy and IA groups (*P* = 0.297).

### 3.2 Plasma concentrations of CD36 and GSH

**Figure 2:**
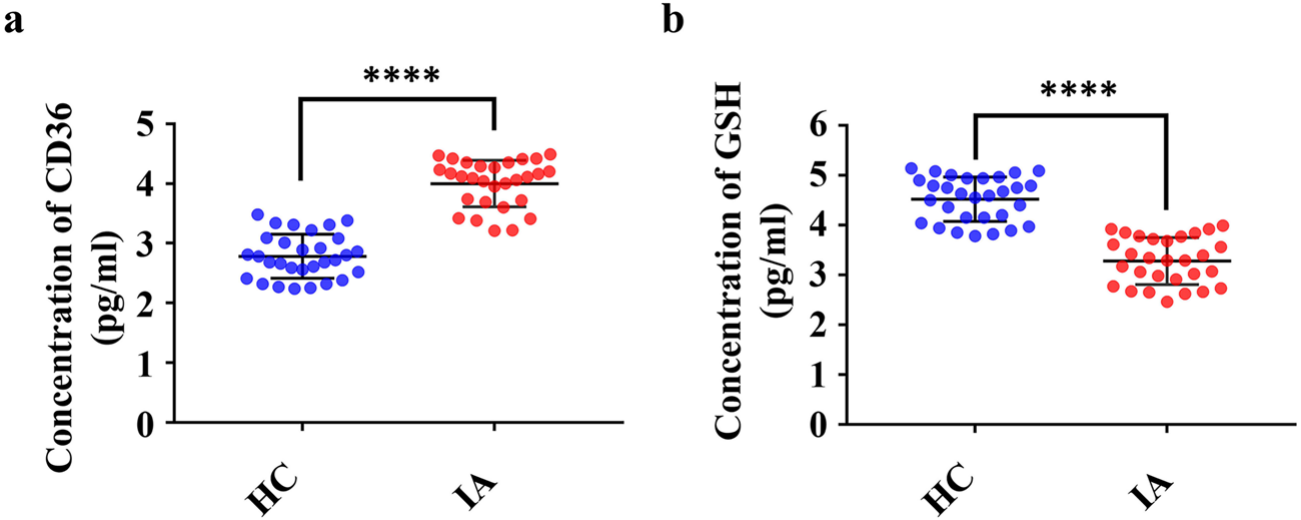
The expression levels of CD36 and GSH in the plasma of IA patients and HCs. (a) Expression levels of CD36 in the plasma of IA patients and HCs. (b) Expression levels of GSH in the plasma of IA patients and HCs. Data are presented as mean ± SD. ****P < 0.0001, *n* = 30 per group.

ELISA analysis was used to measure the CD36 and GSH expression levels in plasma. CD36 and GSH were detected in the plasma of both IA and control groups. The expression level of plasma CD36 in the IA group was significantly higher than that in the HC group, and the difference reached statistical significance (*P* < 0.0001) (Figure 2a). The expression level of plasma GSH in the IA group was significantly lower than that in the HC group (*P* < 0.0001) (Figure 2b). Moreover, no significant difference was reached among patients with different IA locations (Figure S1).

### 3.3 Correlation and ROC curve analysis

**Figure 3:**
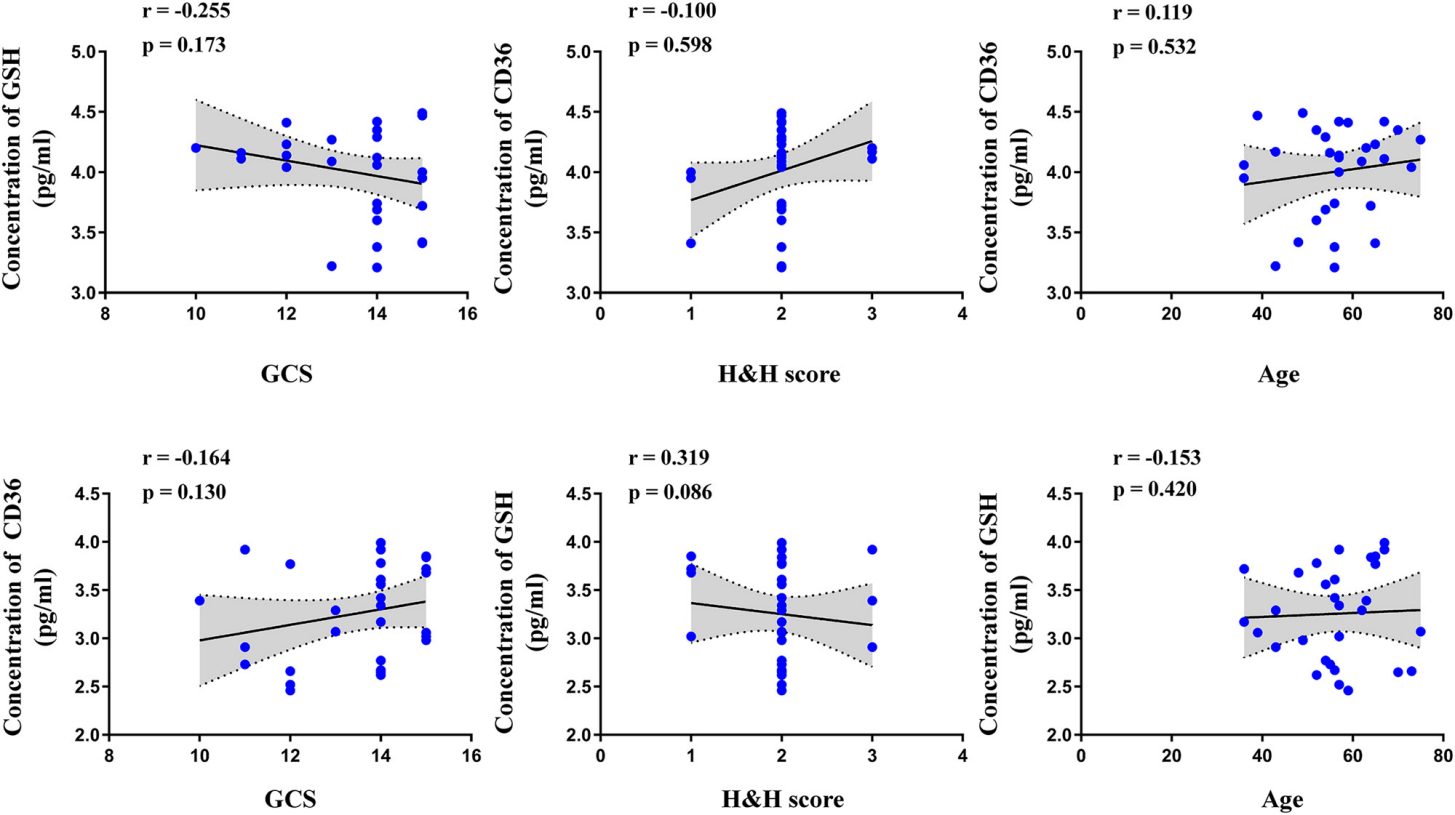
Correlation analysis between the clinical data (patient’s age, GCS, and Hunt–Hess) of IA patients and the expression levels of GSH and CD36.

**Figure 4:**
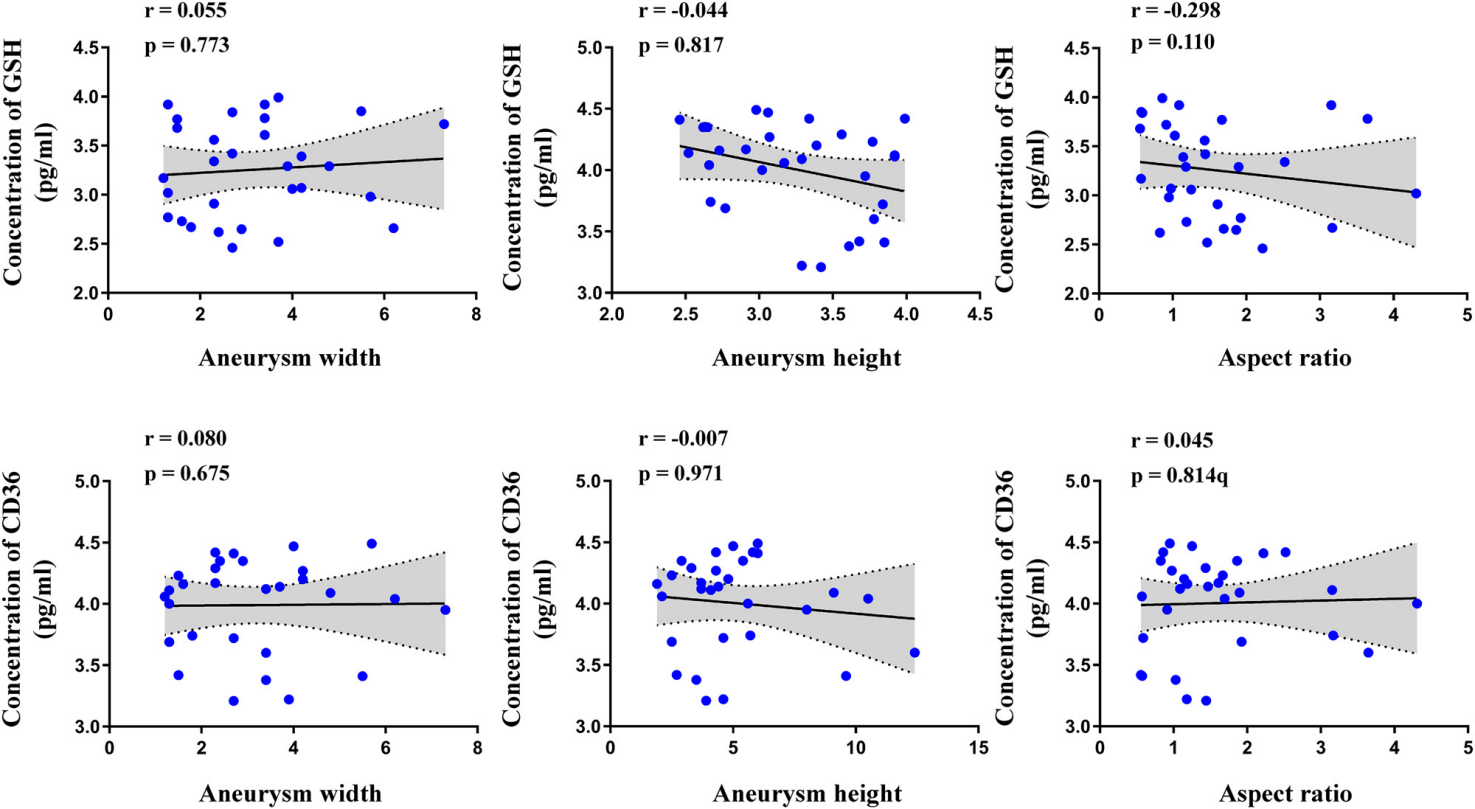
Correlation analysis between the aneurysm parameters (aneurysm size, aneurysm height, aneurysm width, and aspect ratio) of IA patients and the expression levels of GSH and CD36.

**Figure 5:**
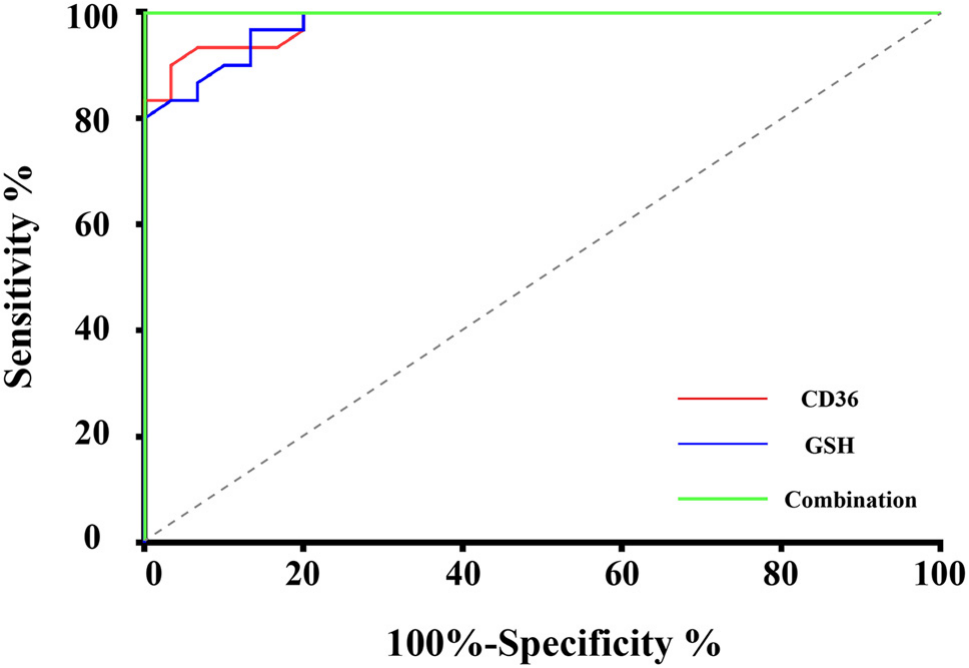
ROC analysis of expression levels of CD36 and GSH in the plasma.

To evaluate the relationship between CD36, GSH, and clinical parameters of IA patients, we analyzed the correlation among the expression levels of CD36, GSH, IA clinical data, and aneurysm parameters. Our results showed no significant correlation between the CD36 expression level and clinical data such as the patient’s age (*P* = 0.420, *r* = −0.153), GCS (*P* = 0.130, *r* = −0.164), and Hunt–Hess (*P* = 0.086, *r* = 0.319) (Figure 3). Also, there was no significant correlation between the CD36 expression level and aneurysm parameters such as the aneurysm size (*P* = 0.928, *r* = −0.017), aneurysm height (*P* = 0.971, *r* = −0.007), aneurysm width (*P* = 0.675, *r* = 0.080), and aspect ratio (*P* = 0.814, *r* = 0.045) (Figure 4). For GSH, there were no significant correlations between the patient’s age (*P* = 0.532, *r* = 0.119), GCS (*P* = 0.173, *r* = −0.255), Hunt–Hess (*P* = 0.598, *r* = −0.100), aneurysm size (*P* = 0.500, *r* = 0.128), aneurysm height (*P* = 0.817, *r* = −0.044), aneurysm width (*P* = 0.773, *r* = 0.055), and aspect ratio (*P* = 0.110, *r* = −0.298) (Figures 3 and 4). Moreover, there is also no significant correlation between aneurysm characteristics, GCS score, and H&H score (Figure S2). The correlation analysis also showed that the expression of GSH significantly correlated with the height of the aneurysm in the middle cerebral artery IA patients (*P* = 0.0415, *r* = 0.6266). Also, the expression of CD36 significantly correlated with the height of the aneurysm in the internal carotid artery IA patients (*P* = 0.0490, *r* = −0.6485) (Figure S3). We further analyzed the sensitivity and specificity of CD36 and GSH for IA prediction using ROC curves. The optimal cutoff point was determined by the ROC curve based on the Youden index. The CD36’s Youden index was 0.867, and the GSH’s Youden index was 0.834. The sensitivity and specificity of CD36 were 90.0 and 96.6%, respectively, and that of GSH was 96.6 and 86.6%, respectively. Moreover, the sensitivity and specificity of CD36 combined with GSH achieved were 100 and 100% (Figure 5).

### 3.4 Evaluation of the logistic regression model

**Figure 6:**
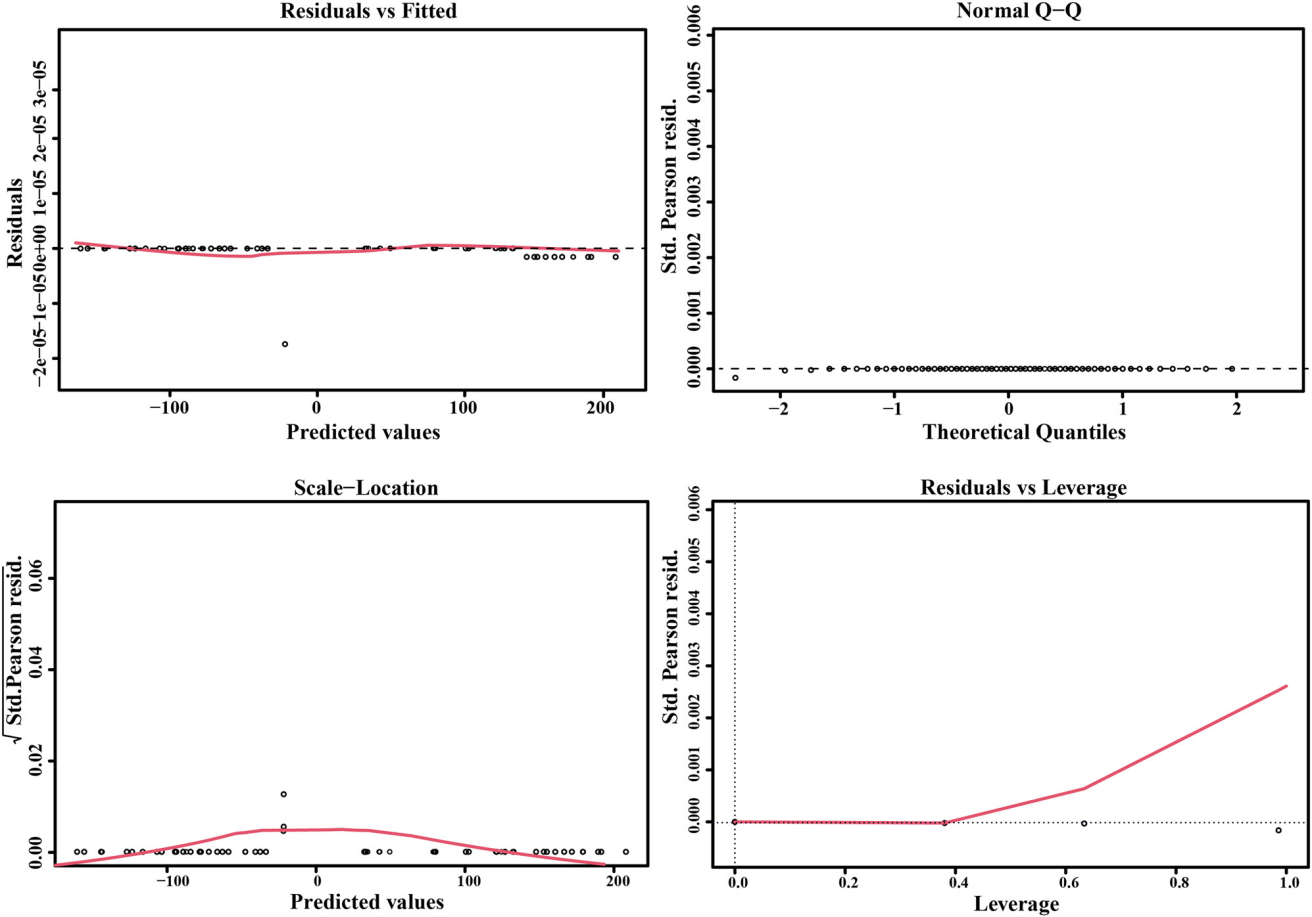
Construction of the logistic regression model. The black dotted line represents the COOK distance, and the points with COOK distance >0.5 were considered as influential points, which would affect the reliability of the model. There were no influential points in the model.

**Figure 7:**
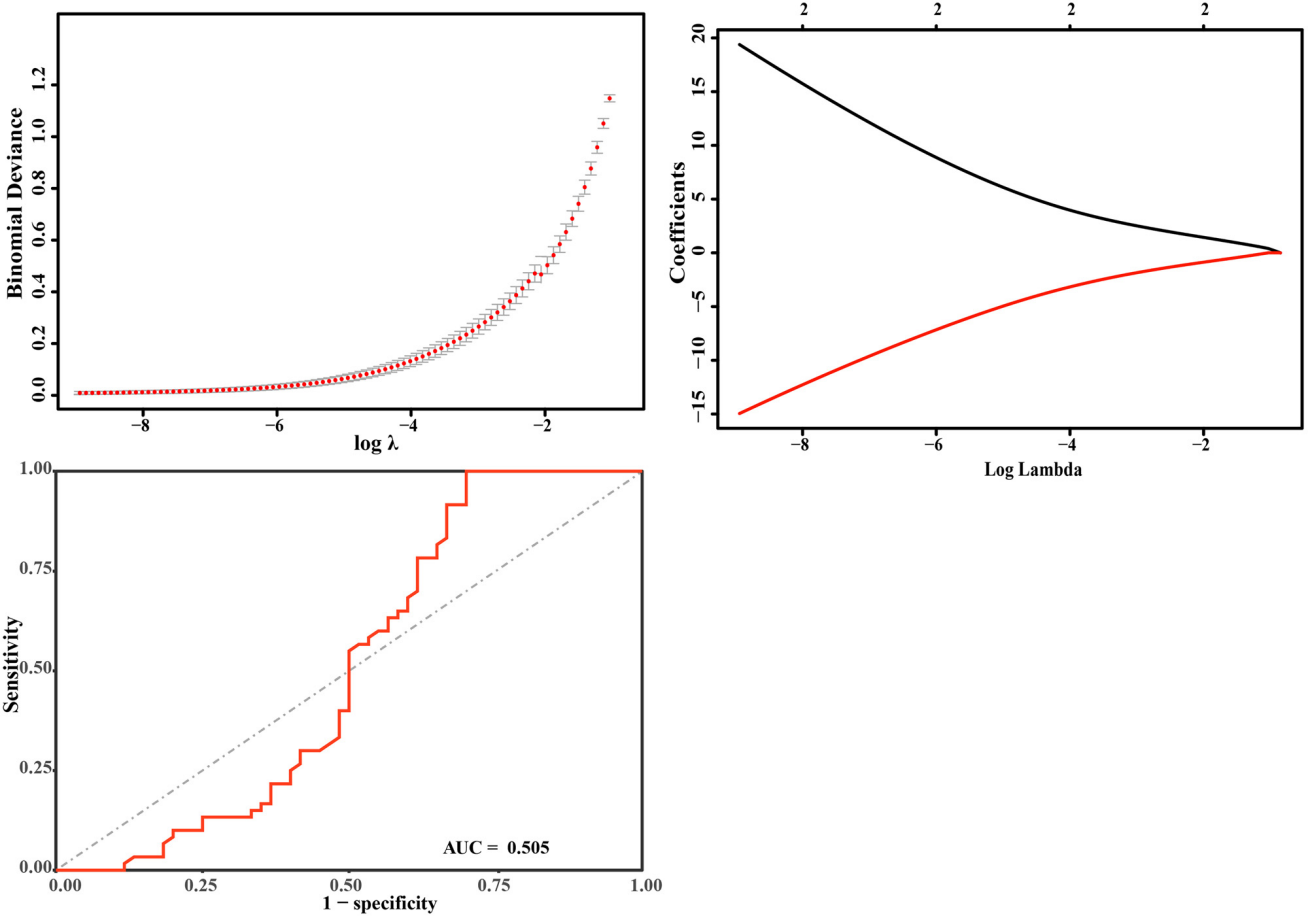
The ROC analysis and cross-validation plot with logistic LASSO regression. For the logistic LASSO regression, self-reported IA was included as the dependent variable, *Y*, and coded as 0 for no IA and 1 for the presence of IA.

The expressions of GSH and CD36 were used as predictive variables. The logistic regression model showed a normal distribution (Figure 6) (Table 2), and the GSH and CD36 values had a good linear relationship with the response variables (clinical data and aneurysm parameters). There are no extreme points that significantly affect the accuracy of the model. The AUC of the logistic model was 0.505, suggesting that the logistic regression model based on GSH and CD36 could reliably distinguish the patients with IA and HCs, and GSH and CD36 could be used as potential biomarkers for the diagnosis of patients with IA (Figure 7).

**Table 2:** Coefficients (standard error) and *P*-values for the multivariate logistic regression

	Estimate	Std. error	*P* value
Intercept	−4.846	8.307	0.56
CD36	5.284	2.128	0.013
GSH	−3.074	1.332	0.021

## 4 Discussion

Although the underlying pathophysiological mechanisms of IA formation and rupture remain unclear, previous studies suggested that vascular endothelial cell inflammation is one of the initiating factors for IA [27]. Oxidative stress, as a significant component in the pathophysiological mechanism of inflammation, also plays a role in the progress of IA formation and rupture [28]. Previous studies have documented that CD36 and GSH were involved in various pathological conditions related to inflammation and oxidative stress [29]. Whether CD36 and GSH could serve as potential biomarkers for IA rupture needs further investigation.

In this study, we aimed to compare the expression levels of plasma CD36 and GSH between patients with ruptured IA and HCs. Further, we evaluated their potential roles as biomarkers for IA rupture. The results showed that plasma CD36 expression was increased in IA patients, while plasma GSH was significantly decreased compared with HCs, confirming our conjecture. The evidence indicated that the pro-inflammatory signaling in vascular endothelial cells could be initially activated by high wall shear stress of blood flow, resulting in abundant macrophages infiltrating the vascular endothelium [30]. Subsequently, macrophages release various cytokines, which further recruit other inflammatory cells, such as neutrophils, T cells, and mast cells [31,27]. CD36 is a scavenger receptor expressed in monocytes, endothelial cells, and platelets, which plays significant roles in mediating lipid uptake, immune recognition, inflammation, molecular adhesion, and apoptosis [32]. CD36 mediates the production of ROS and promotes the occurrence of inflammatory responses by activating the NLRP3 inflammasome [33]. By contrast, CD36 could also upregulate the expression and phosphorylation of focal adhesion kinase, thereby promoting the expression of matrix metalloproteinases (MMPs) [34]. MMPs are proteases most closely related to the formation and rupture of IA. It was reported that MMPs could degrade elastin on the arterial wall, resulting in the loss of the inner elastic layer and weakening of the vessel wall, eventually leading to the rupture of IA [35,36]. Studies have showed that despite the normal lipid levels in IA patients, lipid accumulation, foam cells, and oxidized lipids could be found in both unruptured and ruptured IA walls [37]. Moreover, macrophages infiltrating the vascular endothelium could internalize oxidized low-density lipoprotein (ox-LDL) via CD36 and confine it to the vascular intima, which ultimately leads to the accumulation of vascular wall lipids and the conversion of macrophages to foam cells [38]. The reduction of smooth muscle cells is characteristic of arterial remodeling, which is one of the primary causes of IA formation and rupture [39]. It was also demonstrated that the binding of CD36 on macrophages to ox-LDL could activate the nuclear factor-κB (NF-κB) signaling pathway to trigger an inflammatory response [40]. The NF-κB pathway is a pivotal link in the inflammatory response, and NF-κB-mediated inflammation has been demonstrated to be involved in the pathogenesis of IA [41,42]. Studies have found that the inhibition of NF-κB activity can inhibit the formation and expansion of IA [43]. The above findings indicated that therapeutic targeting CD36-mediated inflammatory responses could be potential candidates for the treatment of IAs.

In addition, inflammation could increase the production of ROS and lead to oxidative stress. ROS could directly increase the expression of inflammatory and adhesion factors, aggravate the inflammatory response, and promote ox-LDL formation [44,45]. Large amounts of ROS deplete antioxidant compounds (GSH, polyphenols, and vitamins) and enzymes in the body, further unbalancing oxidative and antioxidant effects in the body toward oxidation [46]. Among them, GSH is a significant antioxidant that plays a crucial role in maintaining redox homeostasis. GSH-related metabolism is the principal mechanism by which cells protect themselves against oxidative stress [47]. Decreased GSH makes cells more susceptible to oxidative stress, thereby accelerating apoptosis [48]. Studies have shown that oxidative stress can stimulate cells to initiate programmed cell death through an endogenous pathway, resulting in a reduction in the number of cells on the IA wall and a weakening of the vessel wall, which accelerates aneurysm development and rupture [49]. Moreover, oxidative stress can increase CD36 expression, which may be closely related to GSH levels [50]. Studies have found that decreased GSH levels also induced CD36 expression in macrophages and enhanced ox-LDL uptake, thereby promoting lipid accumulation in the vascular wall and foam cell formation [51,52]. Studies have shown that lipid accumulation and foam cell formation are important factors promoting aneurysm wall degeneration, and lipid accumulation in aneurysm walls has been confirmed to be related to aneurysm rupture [53]. The principal sources of macrophages in the walls of IA are monocyte-differentiated macrophages and reduced GSH levels induce monocyte differentiation into macrophages and promote inflammation response [54]. Some studies have found that certain antioxidants restore intracellular GSH levels, which could prevent the transformation of monocytes to macrophages and reduce the expression of CD36, thus playing a protective role [51,54,55]. These findings may provide new therapeutic ideas for antioxidant therapy to inhibit the formation and rupture of IA. Based on our observation, we suggest that CD36 and GSH are involved in the pathological process of inflammation and oxidative stress, which may predispose to rupture and formation of IA. Based on our observation and previous studies, we suggested that the upregulated CD34 and decreased GSH result in an inflammatory and oxidative microenvironment in the blood vessels, and eventually lead to endothelial dysfunction. The altered structure of the vessel wall finally promotes the formation and rupture of IA. This hypothesis needs to be validated by *in vitro* experiments such as using vascular smooth muscle cells.

In order to evaluate the diagnostic values of plasma CD36 and GSH for IA, the ROC curve analysis was performed. The results showed their excellent predictive performance. Previous studies have shown that combining multiple biomarkers could improve diagnostic accuracy than using a single marker [56]. The combination of biomarkers has great potential in identifying diseases, and the improved diagnostic accuracy could even achieve 100% sensitivity and 100% specificity [57]. In this study, the ROC analysis of the combination of two biomarkers showed excellent sensitivity and specificity (each 100.0%), which suggested their potential for serving as biomarkers for IA diagnosis. Furthermore, correlation analysis between the levels of CD36, GSH, and the clinical parameters was conducted to determine their roles in the diagnosis of IA. To our surprise, the results showed that CD36 and GSH had no significant correlation with the GCS score, Hunt–Hess score, age, aneurysm width, aneurysm height, aneurysm neck, and aspect ratio in IA patients. Moreover, logistic regression was used to examine their performance of IA prediction. Our results showed that GSH and CD36 have poor discrimination between IA patients and HCs when clinical characteristics were combined with aneurysm parameters. We speculated that there might be several explanations for these results. First, the study population is too small to represent the predictive values with the logistic regression model [58]. Second, it might be caused by the disease spectrum bias. Clinical studies with a population that lacks diagnostic uncertainty may produce a biased estimate of a test’s performance [59]. Third, our correlation analysis indicated that the expression levels of CD36 and GSH did not correlate with the aneurysm parameters, which implies that they might not participate in the changes in the aneurysm morphology. Evidence also demonstrated a pathogenetic link between hemodynamics and inflammatory response in the development and rupture of IA [60]. As suggested by a previous study, the hemodynamics factor might play a crucial role in the morphologic changes of IA [61]. Here, we suspected that CD36 and GSH might participate in the process of IA formation and rupture but do not affect its morphology. Further, the logistic regression model was established by combining clinical characteristics and aneurysm parameters. Although we evaluated the utility of plasma CD36 and GSH as biomarkers for IA, their diagnostic power deserves further validation in a larger cohort. In addition, IA is a complex disease with both ruptured and unruptured forms. In the future, exploring the expression levels of CD36 and GSH in different subtypes of IA could unravel how these biomarkers change over the course of disease development, providing more valuable insights into their diagnostic and prognostic implications. Investigating the potential roles of CD36 and GSH in IA could offer valuable insights for the development of novel therapeutic strategies and drug targets, providing a stronger foundation for personalized therapy. In conclusion, further study of the role of CD36 and GSH in IA is crucial for the occurrence, development, and treatment of IA, which will provide new insights and directions for the diagnosis, prognosis, and treatment of IA.

This study has some limitations. First, the sample size of this study is small, which may cause biased results to a certain extent. Due to the limited sample size, the enormous difference in the proportion of male and female subjects is also a significant limitation of this study. Evidence has shown that the aneurysm characteristics have a huge difference compared with male and female subjects. In previous studies, women had a higher risk of rupture of IAs than men [62]. Moreover, one previous study has demonstrated that female patients have a greater risk of multiple aneurysms and left internal carotid artery aneurysms, but a lower risk of anterior communicating artery aneurysms [63]. In this study, our results showed no age- and gender-related differences in CD36 and GSH expression. The small sample sizes used in the present study may not provide adequate precision. Moreover, the absence of an unruptured IA group may be another limitation of this study. Previous studies have also demonstrated that aneurysmal subarachnoid hemorrhage might alter the metabolic conditions compared with unruptured IA [64,65]. The unruptured IA group was not included in this study, which may affect the assessment of their diagnostic values as biomarkers. Further sub-group studies (unruptured aneurysm and ruptured aneurysm) with large cohorts are needed in our future work. Second, further investigation of the potential variations within subtypes of IA could provide valuable insights into the heterogeneity of the disease and guide personalized treatment approaches. A previous study reported that cerebral aneurysmal formation depends on their location [66]. In our future work, we hope to carry out another study focusing on the location-dependent difference with a larger sample size. Third, this is an observational study without further animal or *in vitro* cell experiments to understand their underlying mechanisms in the IA pathology process. Furthermore, CD36 knock out (KO) or the GSH metabolism-related gene KO IA mice model could also help in evaluating the role of CD36 and GSH in IA formation. Moreover, we did not measure the expression levels of CD36 and GSH in the aneurysm wall. These limitations are subjects of future work.

In summary, this study revealed a significant increase of plasma CD36 and a decrease of plasma GSH in the patients with ruptured IA compared with HCs. We proposed that CD36 and GSH might be involved in the progress of IA rupture but did not affect the aneurysm morphology. Here, we emphasize the potential of plasma CD36 and GSH as therapeutic targets for the treatment of IA. Subsequent *in vivo* study using a rat model of IA should be carried out to confirm our hypothesis in future work. Moreover, plasma CD36 and GSH could serve as potential biomarkers for IA rupture. Our findings suggest a future role for plasma CD36 and GSH assays in attempting to identify cerebral aneurysms before rupture.

## Supplementary Material

Supplementary material
