# Supplementary material for: Plasma levels of CD36 and glutathione as biomarkers for ruptured intracranial aneurysm
Source: Open Life Sci. 2023 Dec 31;18(1):20220757. doi: 10.1515/biol-2022-0757 (PMC10775171; doi:10.1515/biol-2022-0757)
Supplement: Supplementary material [file biol-2022-0757-sm.pdf]

Supplementary material

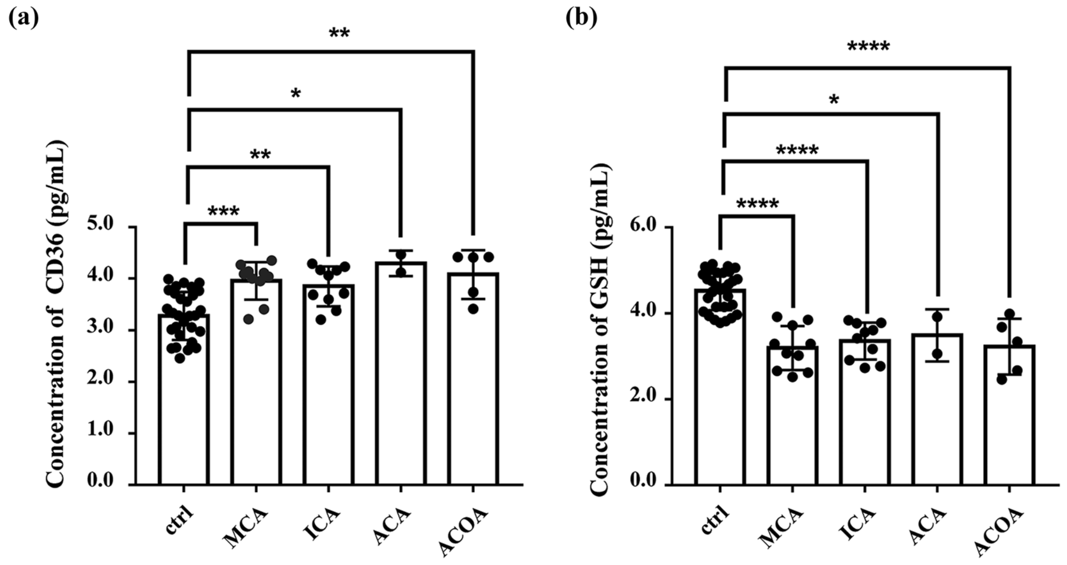

Figure S1: The expression level of CD36 and GSH in plasma of IA patients and healthy controls (ctrl). Middle cerebral artery: MCA, internal carotid artery: ICA, anterior cerebral artery: ACA, Anterior communicating artery: ACOA. *P < 0.05; **P < 0.01; ***P < 0.001; ****P < 0.0001.

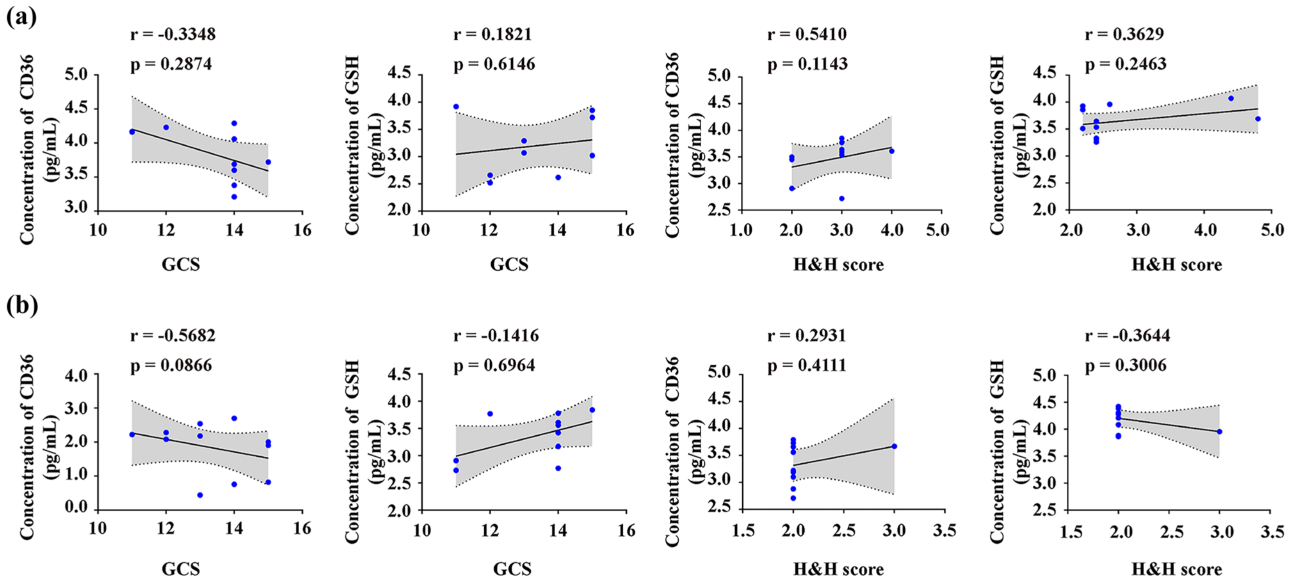

Figure S2: Correlation analysis between the clinical data (patient age, GCS, Hunt-Hess) and the expression levels of GSH and CD36. (a) The correlation analysis of MCA patients. (b) The correlation analysis of ICA patients. Middle cerebral artery: MCA, internal carotid artery: ICA.

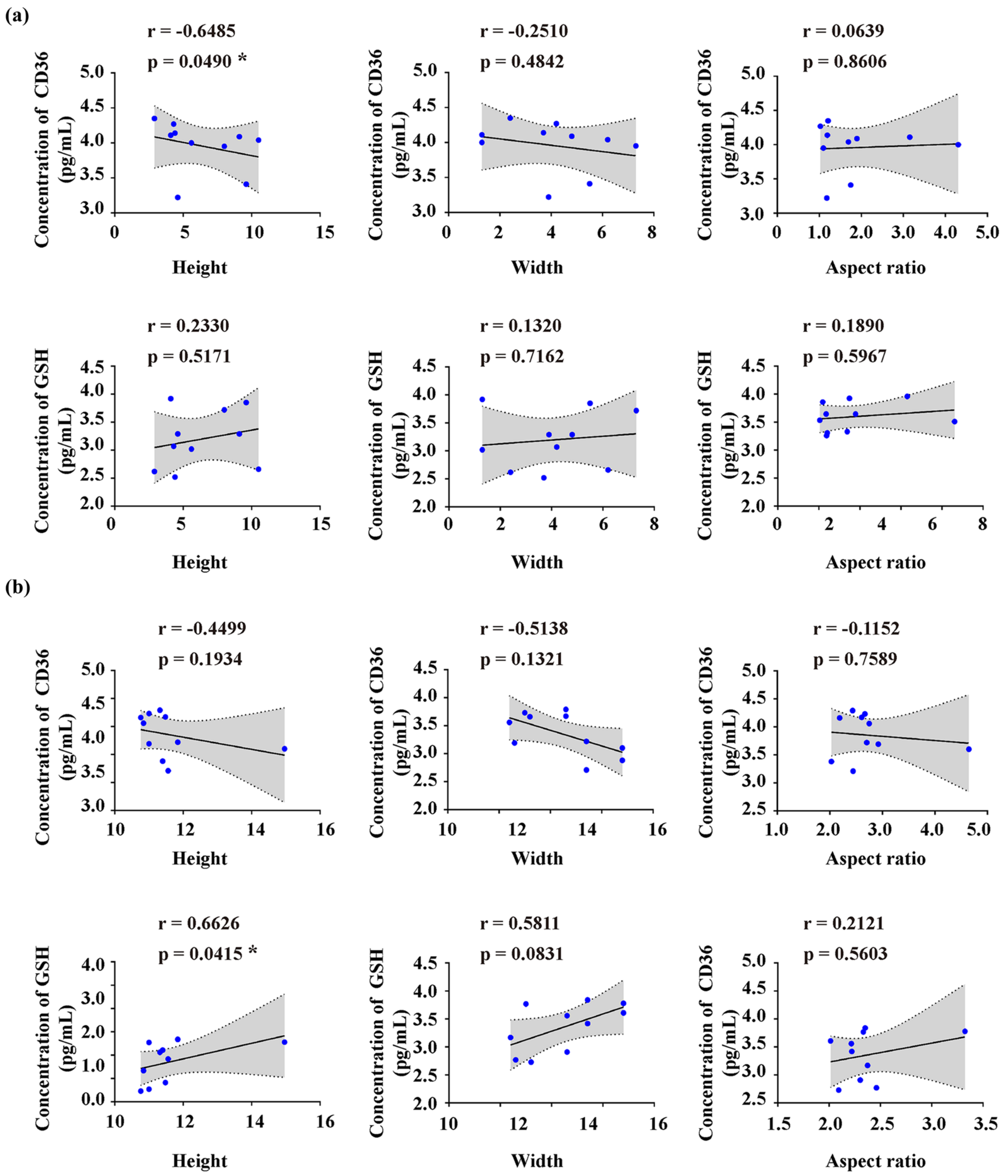

Figure S3: Correlation analysis between the clinical data (patient age, GCS, Hunt-Hess) and the expression levels of GSH and CD36. (a) The correlation analysis of MCA patients. (b) The correlation analysis of ICA patients. Middle cerebral artery: MCA, internal carotid artery: ICA.

Table S1: Normality Tests of clinical parameters

Data	Group	Kolmogorov-Smirnova			Shapiro-Wilk		
		**Statistics**	**Df.**	**Sig.**	**Statistics**	**Df.**	**Sig.**
CD36	IA	0.17	30	0.026	0.902	30	0.009
	Ctrl	0.09	30	200*	0.952	30	0.19
Age	IA	0.113	30	200*	0.967	30	0.458
	Ctrl	0.106	30	200*	0.953	30	0.199
GSH	IA	0.11	30	200*	0.934	30	0.065
	Ctrl	0.128	30	200*	0.92	30	0.026
Aneurysm height	IA	0.195	30	0.005	0.878	30	0.003
Aneurysm width	IA	0.118	30	200*	0.93	30	0.048

*This is a lower bound of the true significance a. a Lilliefors Significance Correction.

IA: Intracranial aneurysm; Ctrl: control.
